# Supplementary material for: Rare earth elements distribution in the river sediments of Ditrău Alkaline massif, Eastern Carpathians
Source: PLoS One. 2024 Dec 5;19(12):e0314874. doi: 10.1371/journal.pone.0314874 (PMC11620583; doi:10.1371/journal.pone.0314874)
Supplement: Attachment — Submitted filename: PONE-D-24-23158_reviewer-comments.docx [file pone.0314874.s001.docx]

Manuscript Number: **PONE-D-24-23158_comments**

Comments

The manuscript having ID PONE-D-24-23158 and entitled “Rare Earth Elements distribution in the river sediments of Ditrău Alkaline Massif, Eastern Carpathians” has focused on the most important environmental geosciences issue. However, the manuscript needs minor revision before recommending for publication in the Journal of PLOS One.

1. Abstract: this section need to be strengthen with prominent results
2. Acronyms need to be explained when first time used. Then use the abbreviation consistently
3. Introduction, Lines 63-65: This part lacks recent background information and needs to be cited with the following most relevant and recent citations:

https://link.springer.com/article/10.1007/s11356-022-24160-9

https://www.sciencedirect.com/science/article/abs/pii/S2352186420316333

<https://doi.org/10.1080/15320383.2024.2306485>

https://doi.org/10.1007/s11368-023-03484-0

4. Justification of this study needs to be strengthened and provide clear objectives
5. Material and methods: sediments sampling need to be explained in details. If the sediments samples were collected in July 2018, then why you wait till 2024 to publish this work?
6. Information about precision and accuracy is missing
7. Results and discussion: This section need to be strengthened with disucssion
